# First person – J. Pablo Sánchez-Ovando

**DOI:** 10.1242/bio.060137

**Published:** 2023-09-14

## Abstract

First Person is a series of interviews with the first authors of a selection of papers published in Biology Open, helping researchers promote themselves alongside their papers. J. Pablo Sánchez-Ovando is first author on ‘ Elevated temperatures increase abnormalities in embryos and reduce larval survival in serpulid polychaetes’, published in BiO. J. Pablo conducted the research described in this article while a master's student in Laboratorio de Sistemática de Invertebrados Marinos (LABSIM)’s lab at Universidad del Mar (UMAR), campus Puerto Ángel, Ciudad Universitaria, Oaxaca, México. J. Pablo is now a PhD student in the lab of Laboratorio de Ecofisiología de Organismos Acuáticos at Centro de Investigación Científica y de Educación Superior de Ensenada (CICESE), Baja California, México, investigating how an increase in temperature and ocean acidification affects marine invertebrates, mainly serpulid polychaetes.


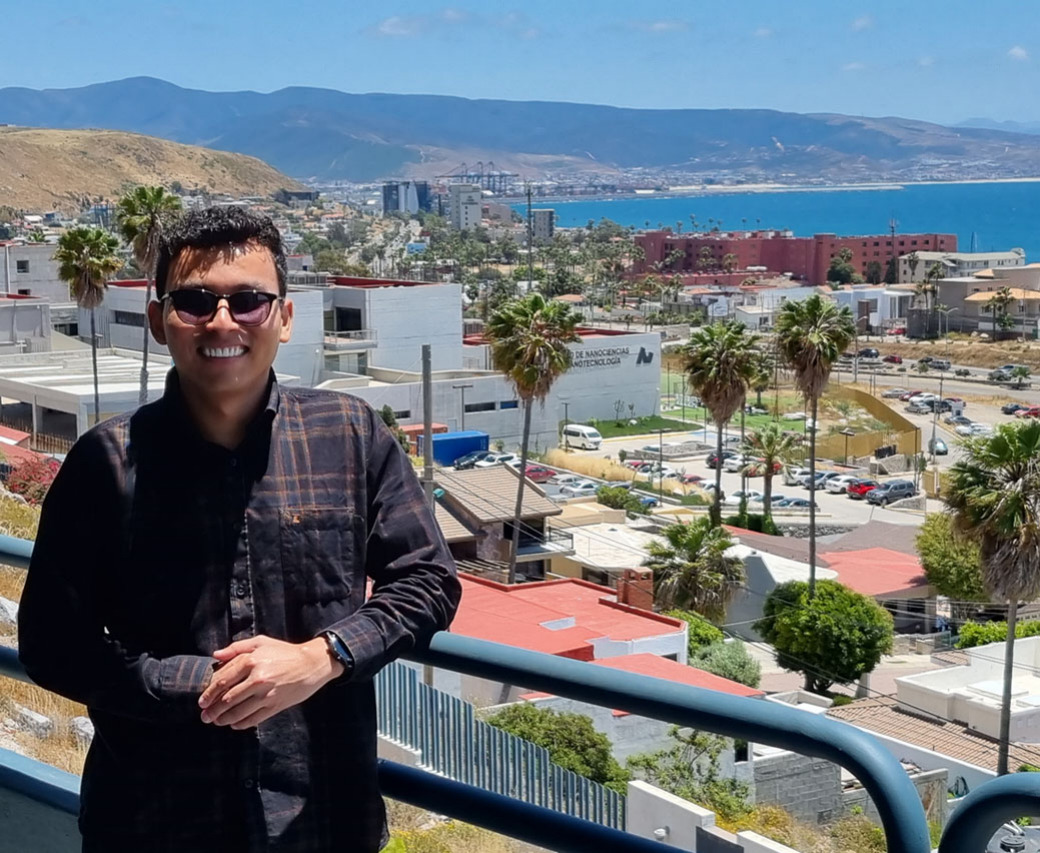


**J. Pablo Sánchez-Ovando**

**Describe your scientific journey and your current research focus**

I began my studies in Marine Biology at the Universidad del Mar (UMAR), Puerto Ángel, Oaxaca, Mexico. In my undergraduate thesis I focused on doing a taxonomic review of serpulid polychaetes of the genus *Spirobranchus* from tropical America. Later, at the same university, I completed my master's studies in Marine Ecology, investigating the effect of increased temperature on the embryos and larvae of two Spirobranchus species. Currently, I am studying a PhD in Life Sciences with a focus on Marine Biotechnology at the Centro de Investigación Científica y de Educación Superior de Ensenada (CICESE), B.C., México. My current project is focused on understanding the effects of increasing temperatures and ocean acidification on the physiological response of *Spirobranchus spinosus*, a temperate water species.

**Who or what inspired you to become a scientist?**

Beginning my undergraduate studies in Marine Biology, which is a scientific career, led me to make the decision to continue with my academic studies, and to be a scientist in the near future. In addition, during my undergraduate degree at Universidad del Mar, I was always surrounded by excellent professors, particularly Dr Rolando Bastida-Zavala and Dr Francisco Benítez-Villalobos, who saw something in me and believed that I had the potential to become an excellent scientist.

**How would you explain the main finding of your paper?**

Increases in temperature (32-34°C) negatively affected the embryos and larvae of the two species of sessile marine worms. This is very serious because the only way these worms can move is during their larval stage. In the near future, under a global warming scenario, the distribution of both species could be modified, causing a loss of biodiversity, changes in the trophic chain, and alterations in the water column, such as excess organic matter.

**Figure:**
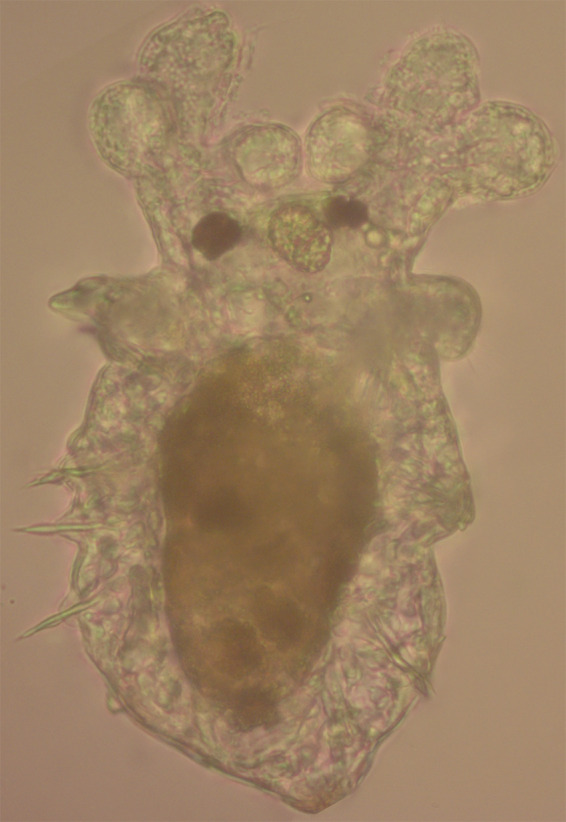
***Spirobranchus incrassatus* larva in metamorphosis 21 days post-fertilization (reared at 28°C).** It is possible to observe branchial rudiments, eye spots, the stomach, and three pairs of chaetae.

**What are the potential implications of this finding for your field of research?**

With this finding, the next step is to carry out more studies of this type with other species of marine invertebrates to determine if they will respond in the same way as these two species of serpulid polychaetes. With this information, it will be possible to generate a broader panorama to understand what will happen to the different populations of marine invertebrate species in the study region.

“…the next step is to carry out more studies of this type with other species of marine invertebrates to determine if they will respond in the same way as these two species of serpulid polychaetes.”

**What do you enjoy most about being an early-career researcher?**

What I enjoy the most is that I am having the complete freedom to carry out the investigations that interest me. I enjoy every field trip and the hours I spend in the laboratory doing my experiments. I enjoy being able to see living organisms in the laboratory and take care of them. However, what I like most is sharing my research findings.

**What piece of advice would you give to the next generation of researchers?**

I would tell them: You do not give up meeting your goals. Always do what excites you the most. I want you to know that the path of science is not easy, but it is fun, and each achievement feels very rewarding.

**What's next for you?**

In the next two years, I will complete my doctoral studies. Subsequently, I would like to do a postdoctoral stay abroad in order to travel a lot, continue publishing the results of my research, and meet other researchers with whom I can collaborate.
